# Complete genome sequence of polyurethane-degrading *Aeromicrobium* sp. JP4

**DOI:** 10.1128/mra.00150-26

**Published:** 2026-05-12

**Authors:** Junbin Ji, Jiayu Gao, Yazhou Bao, Xiaoli Li, Ru Zhao, Weiduo Wang, Liangui Tan, Changqing Zhu, Xianghua Wu, Yulong Wu, Like Wang, Ailing Ben, Tingli Liu

**Affiliations:** 1 Nanjing Key Laboratory of Quality and Safety of Agricultural Products, College of Food Science, Nanjing Xiaozhuang University 74587, Nanjing, Jiangsu, People's Republic of China; University of Southern California, Los Angeles, California, USA

**Keywords:** polyurethane plastic, biodegradation, *Aeromicrobium* sp. JP4

## Abstract

*Aeromicrobium* sp. JP4, an efficient degrader of polyurethane plastic, was isolated from a plastic waste dumpsite of Nanjing City, Jiangsu Province, China. We report the complete genome sequence of strain JP4, which consists of a circular chromosome of 3,493,617 bp.

## ANNOUNCEMENT

Polyurethane plastics play an important role in our lives, but their waste causes serious pollution problems (1, 2). Our clarification of the genome sequence of the polyurethane-degrading *Aeromicrobium* sp. JP4 is expected to provide insights into the mechanisms of polyurethane degradation and help its application in the treatment of polyurethane plastic waste in the future.

*Aeromicrobium* sp. JP4 was isolated from a plastic waste dumpsites of Nanjing City, Jiangsu province, China (32°01′08″ N, 118°90′77″ E). One gram of soil sample was suspended in 100 mL of sterile water, serially diluted, and then plated on DLN medium agar for incubation at 30°C for 5 days. The DLN medium was prepared using mineral salt medium (MSM) (3), supplemented with 0.5% Impranil DLN W 50 (wt/vol) and 5% lysogeny broth (LB) (vol/vol) (4), and adjusted to pH 7.0. Impranil DLN W 50 is a colloidal polyester polyurethane (5), which was purchased from Covestro AG Co., Ltd. (Shanghai, China). Strain JP4 was selected based on its ability to form a clear halo on DLN medium agar at 30°C. Genomic DNA extracted from cultures grown in LB at 30°C for 24 h with agitation (180 rpm) using the FastPure Bacteria DNA Isolation Mini Kit (Vazyme, Nanjing, China) was used for both Nanopore and Illumina library preparation. The isolate was identified by 16S rRNA gene clone sequencing with 27F (5′-AGAGTTTGATCCTGGCTCAG-3′) and 1492R (5′-GGTTACCTTGTTACGACTT-3′) primers. Based on BLASTN analysis, strain JP4 (16S rRNA gene: PX957052) showed the highest similarity (99.93%) to *Aeromicrobium phoceense* Marseille-Q0843^T^ and more than 96% similarities to other species of *Aeromicrobium*, indicating that strain JP4 was a member of the *Aeromicrobium*. The complete genome of *Aeromicrobium* sp. JP4 was generated using Illumina Novaseq 6000 platform and Oxford Nanopore PromethION (Wuhan Benagen). For Illumina sequencing, VAHTS Universal Plus DNA Library Prep Kit (Vazyme, China) was used for library construction. Sequencing was carried out using the Illumina NovaSeq 6000 platform with the paired-end mode (2 × 150 bp). Raw reads were processed with fastp v0.23.2 (6) to remove adapters, trim low-quality bases, and discard short fragments, yielding 14,751,534 clean reads. For PromethION sequencing, a ligation-based library (SQK-LSK110) was prepared from 1 µg of unsheared genomic DNA (>1 kb) and sequenced on an R9.4.1 flow cell. The data were generated and underwent quality control using Guppy in fast mode version 5.0.16 (7), which filtered out low-quality fail reads, resulting in 181,180 high-quality reads with an *N_50_* length of 14,185 bps. Hybrid *de novo* assembly was performed with Unicycler v0.5.0 (8), which identified overlaps, trimmed contig ends, and confirmed circular topology without genome rotation. The assembly yielded a complete circular chromosome (3,493,617 bp), verified by overlapping ends. The average Illumina and Nanopore coverages were 628× and 404×, respectively. The genome was annotated with the NCBI Prokaryotic Genome Annotation Pipeline (PGAP) version 6.10 (9–11). Default parameters were used for all software unless otherwise specified. Relevant statistics are listed in Table 1.

**TABLE 1:** Genomic features of *Aeromicrobium* sp. JP4

Feature	Value
No. of Illumina clean reads	14,751,534
No. of Nanopore clean reads	181,180
Total no. of Illumina bases	2,204,328,668 bp
Total no. of Nanopore bases	1,412,159,002 bp
Genome coverage (Illumina; Nanopore)	628×; 404×
Chromosome size	3,493,617 bp
GC content	70.48%
Total no. of genes	3,451
No. of coding sequences	3,378
No. of pseudogenes	19
No. of rRNAs	6
No. of tRNAs	45

## Data Availability

The genome sequence of *Aeromicrobium* sp. JP4 has been deposited in the GenBank database under accession number JBUDIG000000000. The BioProject and BioSample accession numbers are PRJNA1417329 and SAMN54994422. The raw sequencing data were deposited in the Sequence Read Archive (SRA) database under accession numbers SRR37096166 and SRR37117119.
